# Incarceration of the Afferent Loop into the Sutured Closed Mesenteric Defect after Gastrectomy Followed by Billroth-II Reconstruction for Gastric Cancer: Two Case Reports

**DOI:** 10.70352/scrj.cr.25-0167

**Published:** 2025-05-29

**Authors:** Kiyotomi Maruyama, Tadaaki Shimizu, Kou Shimada, Arano Makino, Natsuhiro Morita, Tasuku Kawaguchi, Takahiro Amano, Tomoki Shirota, Kuniyuki Gomi, Motohiro Mihara

**Affiliations:** Department of Surgery, Suwa Red Cross Hospital, Suwa, Nagano, Japan

**Keywords:** Billroth-II reconstruction, internal hernia, mesenteric defect, gastric cancer, laparoscopic surgery

## Abstract

**INTRODUCTION:**

Internal hernia is a critical complication after laparoscopic gastrectomy with Roux-en-Y, Billroth-II or double tract reconstruction. It is recommended that mesenteric defects should be closed to prevent internal hernias. We reported two patients who developed internal hernias, in which the afferent loop of Billroth-II reconstruction became incarcerated into the closed mesenteric defects.

**CASE PRESENTATION:**

A man in his late 40s had undergone laparoscopic distal gastrectomy 3 months prior for gastric cancer followed by Billroth-II reconstruction, in which mesenteric defect was sutured closed. The patient visited our hospital complaining of sudden severe upper abdominal pain and was diagnosed with afferent loop obstruction due to an incarcerated internal hernia complicated by acute pancreatitis. Emergency surgery, in which intestinal incarceration was relieved and intestinal ischemia was not found, was performed on the same day as admission. However, postoperative duodenal microperforation occurred, making treatment difficult. A woman in her late 70s had undergone laparoscopic distal gastrectomy 7 days prior for gastric cancer followed by Billroth-II reconstruction, in which mesenteric defect was sutured closed. The patient complained of nausea without abdominal pain and was diagnosed with afferent loop obstruction due to an incarcerated internal hernia. Emergency surgery, in which intestinal incarceration was relieved and intestinal ischemia was not found, was performed on the same day. The patient was discharged uneventfully. In both cases, a hernia orifice formed in the Treiz ligament area, and the afferent loop was incarcerated into the closed mesenteric defect.

**CONCLUSIONS:**

Incarcerated internal hernias should be treated as soon as possible. Although closure of the mesenteric defects after Billroth-II reconstruction is necessary to prevent internal hernias, mesenteric defects should be closed on the left side as far away from the Treiz ligament as possible.

## Abbreviations

B-II
: Billroth-II reconstruction
GC
: gastric cancer
HP
: Helicobacter pylori
IH
: internal hernia
MD
: mesenteric defect
R-Y
: Roux-Y

## INTRODUCTION

Despite substantial declines in noncardia GC incidence due mainly to the global trend in decreasing HP infection rates, the incidence of proximal gastric cancer, that is, adenocarcinoma of the esophagogastric junction or upper portion of the stomach, has been rising markedly worldwide,^1–4)^ including in Japan.^5)^ For upper gastric cancer, total gastrectomy, proximal gastrectomy, or distal gastrectomy is selected depending on the location and progression of the cancer. As the number of upper gastric cancers is increasing, B-II and R-Y reconstruction may become more common for distal gastrectomy than Billroth-I reconstruction. In reconstructions such as B-II and R-Y, which leave an MD, postoperative IH can be a problem. Nonclosure of MDs, the laparoscopic approach, and the totally laparoscopic approach were found to be independent risk factors for IH.^6)^ MD should be closed to prevent IH after gastrectomy.^6,7)^

IHs associated with the reconstructed intestine after laparoscopic gastrectomy was observed in 2.0% of cases (R-Y: one case; double tract: one case; B-II: four cases; including cases in which the MDs were not closed). Initially, we were not closing the MDs after B-II, when we were performing open gastrectomy. We started closing MDs after our first experience with an IH after gastrectomy followed by B-II, in which the MD was not closed. However, we experienced two cases of IH in which the afferent loop of B-II became incarcerated into the closed MD despite the suture closure. The mechanisms are reported in a literature review. There have been no reports of the intestine being incarcerated into the site of MD suture closure.

## CASE PRESENTATION

### Case 1

**Fig. 1:**
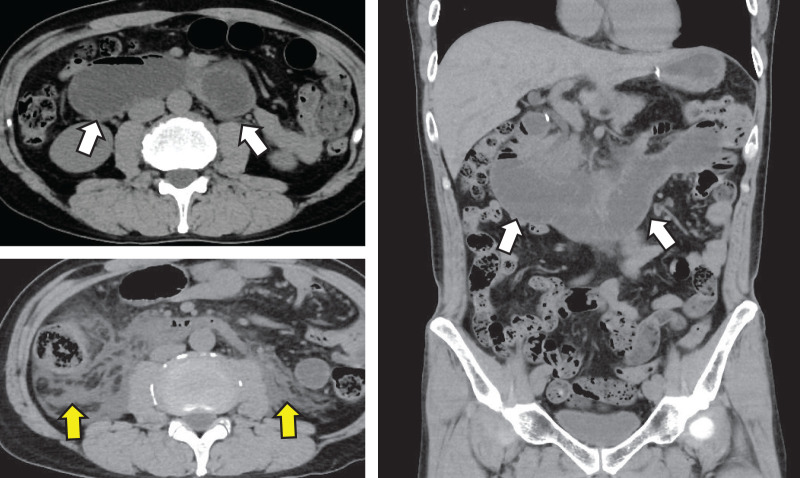
CT scan revealed abnormal dilation of the intestine from the duodenum to the afferent loop (white arrow) and widespread edema in the right retroperitoneum posterior to the duodenum (yellow arrow).

A man in his late 40s visited our emergency room late at night, complaining of sudden severe epigastric pain, and was admitted. The patient had undergone laparoscopic distal gastrectomy followed by B-II with antiperistalsis anastomosis through the antecolic route, in which the MD was sutured closed with continuous suturing with a 3-0 nonabsorbable barbed suture, for pStage I GC 3 month prior. The patient had no other medical history. The patient’s height was 177.7 cm, his weight was 70.0 kg, his body temperature was 36.2°C, his blood pressure was 139/103 mmHg, his pulse rate was 55 beats/min, and his respiratory rate was 18 breaths/min. Physical examination revealed that the abdomen was flat and soft, but there was tenderness in the epigastrium and no rebound tenderness or muscular guarding. Laboratory examination revealed no abnormalities except for a high serum amylase value of 991 U/L. A CT scan revealed abnormal dilation of the intestine from the duodenum to the afferent loop, and widespread edema around the pancreas head and in the right retroperitoneum posterior to the duodenum (**Fig. 1**). The patient was diagnosed with afferent loop obstruction due to an incarcerated IH complicated by acute pancreatitis.

**Fig. 2:**
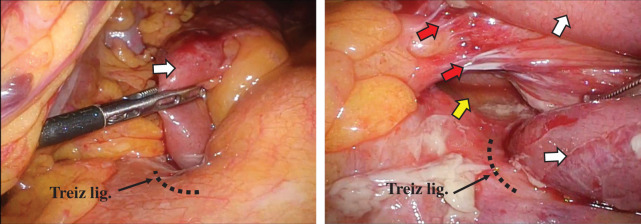
The incarcerated afferent loop (white allow) was carefully and gently returned to its original position. It was found after hernia reduction that a hernia orifice (yellow arrow) formed between the lower end of the suture line (red arrow) in the initial operation and the Treiz ligament.

We performed emergency surgery 10 hours after his visit. The surgery was performed laparoscopically. Surgical findings revealed that the afferent loop was incarcerated at the lowest end of the suture line in the MD that had been closed during the initial operation and that the duodenum and the incarcerated afferent loop were significantly dilated. The intestine was carefully and gently returned to its original position (**Fig. 2**). We decided not to close the hernia orifice because it was close to the duodenum at the Treiz ligament. The operation lasted for 85 minutes, and the blood loss volume was 5 g. In the postoperative course, on the 8th postoperative day, a duodenal microperforation occurred, and emergency surgery, involving peritoneal lavage, abdominal drainage, retrograde duodenal tube drainage, and biliary drainage, was performed. The patient had difficulty with treatment and was discharged on the 59th postoperative day.

### Case 2

**Fig. 3:**
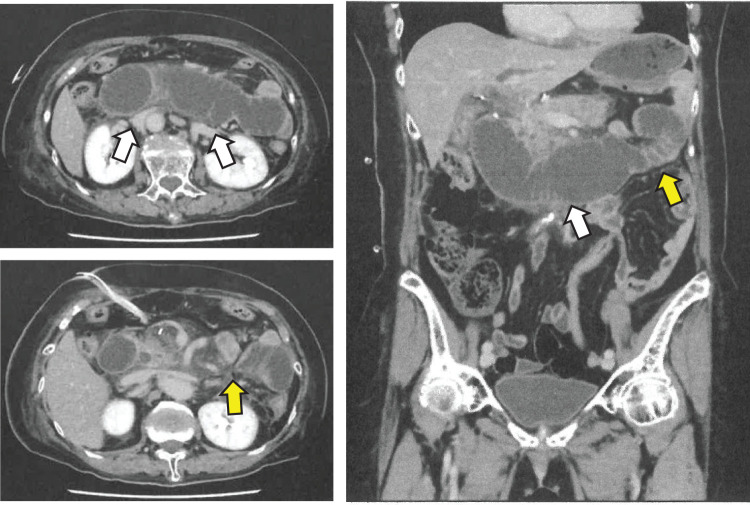
CT scan revealed abnormal dilation of the intestine from the duodenum to the afferent loop (white arrow) and stricture after the dilatation (yellow arrow).

A woman in her late 70s experienced nausea without abdominal pain on the 7th postoperative day. The patient had undergone laparoscopic distal gastrectomy followed by B-II with antiperistalsis anastomosis through the antecolic route, in which the MD was sutured closed with continuous suturing with a 3-0 nonabsorbable barbed suture, for pStage IIA GC 7 days prior. The patient’s medical history included hypertension. The patient’s height was 150.3 cm, her weight was 58.0 kg, her body temperature was 37.2°C, her blood pressure was 147/74 mmHg, her pulse rate was 93 beats/min, and her respiratory rate was 168 breaths/min. Physical examination revealed that the abdomen was flat and soft without tenderness. Laboratory examination revealed a high level of inflammatory response, with a WBC count of 135.9 × 10^2^/μL and a CRP value of 19.2 mg/dL, hypoproteinemia with an Alb value of 2.7 mg/dL; and an increase in hepatobiliary data, with an AST value of 310 IU/L, an ALT value of 433 IU/L, a total bilirubin value of 10.57 mg/dL, an LDH value of 357 IU/L, an ALP of 318 IU/L, and a γGTP value of 436 U/L, but no other abnormalities were found. A CT scan revealed abnormal dilation of the intestine from the duodenum to the afferent loop and stricture after the dilatation (**Fig. 3**). The patient was diagnosed with afferent loop obstruction due to an incarcerated IH.

**Fig. 4:**
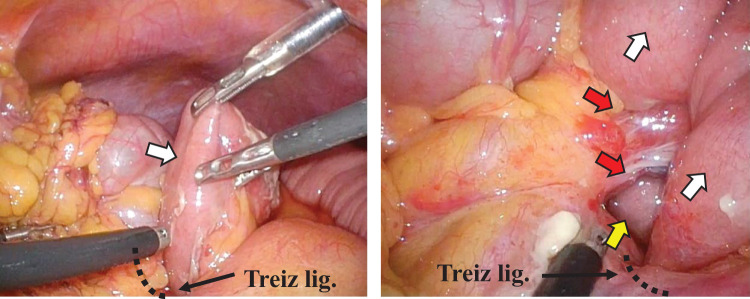
The incarcerated afferent loop (white allow) was carefully and gently returned to its original position. It was revealed after hernia reduction that a hernia orifice (yellow arrow) formed between the lower end of the suture line (red arrow) in the initial operation and the Treiz ligament.

We performed emergency surgery that same day. The surgery was performed laparoscopically. Surgical findings revealed that the afferent loop was incarcerated at the lowest end of the suture line in the MD that had been closed during the initial operation and that the duodenum and the incarcerated intestine were significantly dilated. The intestine was carefully and gently returned to its original position (**Fig. 4**). We decided not to close the hernia orifice because it was close to the duodenum at the Treiz ligament. The operation lasted for 56 minutes, and the blood loss volume was 3 g. The patient’s postoperative course was uneventful, and the patient was discharged on the 20th postoperative day.

## DISCUSSION

In Japan, the prevalence of HP infection has decreased^8)^; consequently, the incidence of HP-related GC has decreased. However, the incidence of HP infection-negative GC is expected to increase. Imamura et al.^9)^ reported that HP infection-negative GC lesions were predominantly observed in the fundic gland (72.2%). For these reasons, an increase in GC in the upper part of the stomach is predicted in the near future, which may result in an increase in total gastrectomy, proximal gastrectomy, and distal gastrectomy with a small stomach remnant, leading to an increase in R-Y, double tract reconstruction, and B-II.

IH is a well-known complication following laparoscopic and open gastrointestinal surgery and is a critical problem because it can cause life-threatening conditions, such as bowel strangulation or perforation.^10,11)^ IH is particularly likely to occur after surgery in which MDs are formed, such as those followed by R-Y and B-II, or after surgery in which the intestine is fixed to the abdominal wall, such as jejunostomy or colostomy. Low level of postoperative intestinal adhesion is a risk factor for the development of IH. Free intestines can become incarcerated in the MD or become entangled with the fixed intestine. IH, which obstructs the duodenum in the reconstructed intestine, is often complicated by acute pancreatitis, especially when the duodenum is blind-ended, and requires immediate treatment. Laparoscopic surgeries have long been the standard treatment for gastrointestinal cancer surgery, and the use of adhesion prevention agents has also become widespread.^12)^ These procedures and agents are less invasive and reduce the risk of postoperative adhesions. In subsequent operations following laparoscopic surgery, surgeons notice less adhesion than they do after conventional open surgery. Laparoscopic surgery offers various benefits to patients, but the low incidence of postoperative intestinal adhesions appears to increase the risk of IH.

Kang et al.^6)^ analyzed 111 (1.7%) patients with IHs identified via CT or surgical exploration and reported that among the 6474 patients who underwent gastrectomy for GC, 2.0% developed IHs after laparoscopic surgery, 0.9% developed IHs after open surgery, and 0%, 1.1%, 3.1%, and 2.3% developed IHs after undergoing Billroth-I reconstruction, B-II, R-Y, or double tract, reconstruction, respectively. IH occurs most often after R-Y, the so-called Petersen’s hernia. According to the multivariate analysis, nonclosure of MDs (P < 0.01), laparoscopic approach (P < 0.01), and totally laparoscopic approach (P = 0.03) were independent risk factors for IH. The importance of closing MDs was emphasized to prevent IH after gastrectomy. We have been closing the MDs with sutures since our first experience with IH after B-II.

The median interval time to the development of IH was reported to be 450 days after gastrectomy.^6)^ It is presumed that stitches closing the MDs may loosen or that gaps between sutures may become larger, as the amount of mesenteric fat tissue decreases due to excess body weight loss after gastrectomy. This may contribute to the development of IHs even after closure of the defects.^13–15)^ There is a high possibility of dehiscence, especially if the suture is absorbable. However, in our cases, MDs were sutured with non-absorbable sutures and IHs developed at 7 days and 3 months immediately after gastrectomy, so this is not the only cause of incarceration of the afferent loop.

**Fig. 5:**
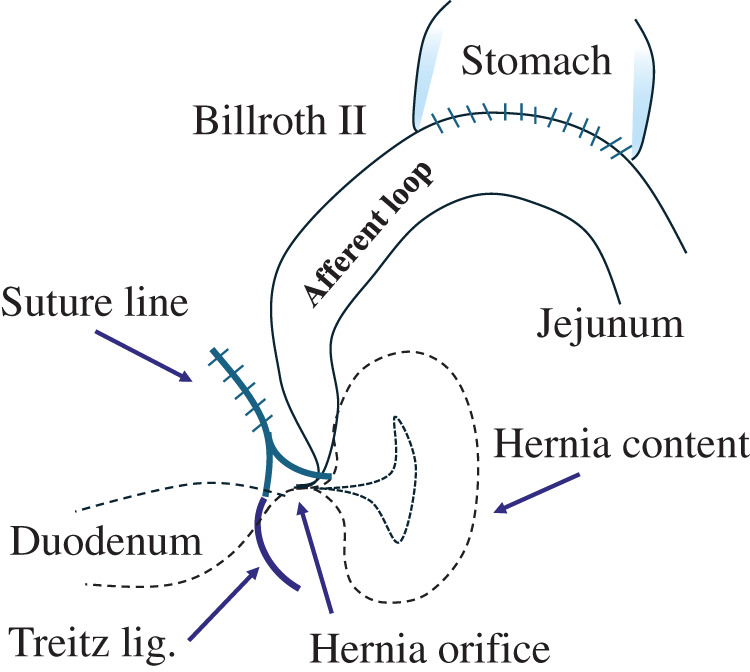
This schema shows the mechanism of IH after Billroth II. As a result of closure of the MD adjacent to the Treiz ligament, a hernia orifice was formed at the site. The duodenum at the Treiz ligament was pulled dorsally and the afferent loop was retracted into the hernia orifice.

**Fig. 6:**
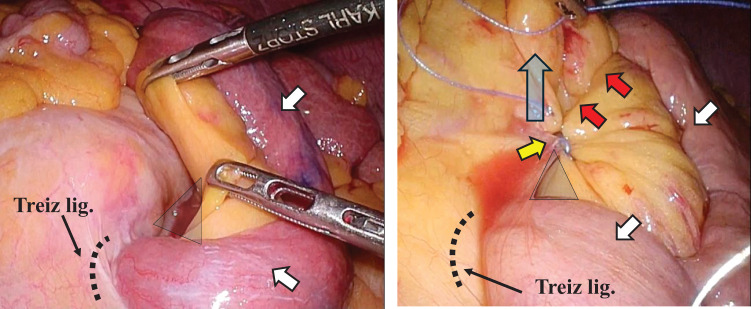
Surgical findings in the initial operation in case 1. Afferent loop (white arrow). The mesenteric defect was continuously closed using a nonabsorbable barbed suture, with the starting point (yellow arrow) close to the Treiz ligament and the proceeding suture toward the gastric remnant (red arrow). This method involved shortening the suture line (red arrow) and lifting upward the sutured mesenterium (blue arrow), resulting in the formation of a hernia orifice (triangle) close to the Treiz ligament.

**Fig. 7:**
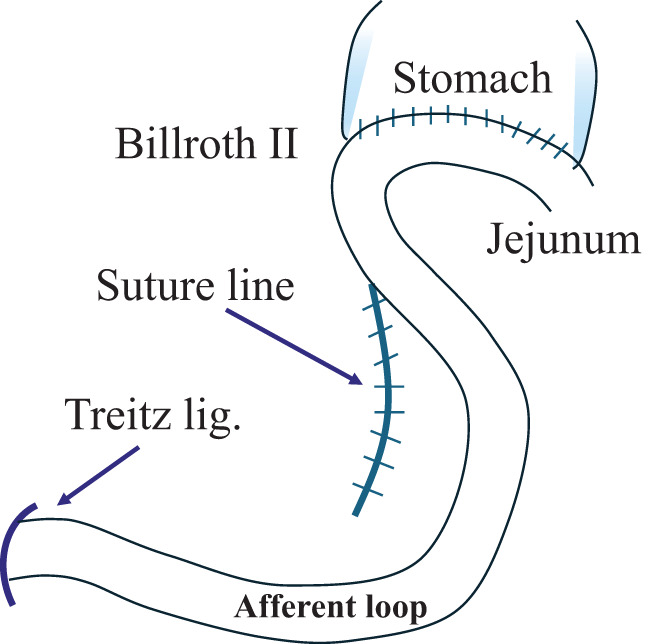
To prevent the development of the IH, the MD should be closed with the starting point of the suture and the suture line being at the left side as far as possible from the Treiz ligament.

Left paraduodenal hernia, a rare condition, has been reported.^16–18)^ Left paraduodenal hernias occur when the intestinal tract prolapses to the dorsal side of the descending mesocolon via the dorsal side of the inferior mesenteric vein (Landzert’s fossa). This fossa is formed congenitally, and although the anatomical structure that forms the hernia orifice is completely different, we believe that a similar phenomenon occurred in our patients. In other words, the terminal part of the duodenum at the Treiz ligament, which was fixed to the retroperitoneum, became the leading part, passed through a hernia orifice formed at the Treiz ligament, protruded into the dorsal side of the mesenterium, and the following afferent loop was pulled in and became incarcerated (**Fig. 5**). Furthermore, in our patients, the MDs were continuously closed using a barbed suture, with the starting point close to the Treiz ligament and the proceeding suture toward the gastric remnant. This method involved shortening the suture line and lifting upward the sutured mesenterium, resulting in the formation of a hernia orifice close to the Treiz ligament, which was fixed to the retroperitoneum (**Fig. 6**). Therefore, the MD should be closed with the starting point of the suture and the suture line being at the left side as far from the Treiz ligament as possible (**Fig. 7**). In this regard, performing MD suturing following B-II with isoperistalsis may be effective since suturing can be easily performed without involving the Treiz ligaments. It is thought that continuous suturing will not be an issue if the suture line is placed away from the Treiz ligament. The precautions regarding the position of the suture line also apply to R-Y reconstruction.

One point to consider is whether the hernia orifice should be sutured closed. We did not dare close the hernia orifice in our patients. In fact, since we had no experience with this type of IH, we were unable to elucidate its mechanism during the operation. We were aware that the duodenum at the Treiz ligament was pulled dorsally and that this intestine was the leading part, so we were worried that closing the hernia orifice would cause further stenosis and result in the duodenum becoming incarcerated at that site again. The cause of the IH in these cases was that the closure of the MD was performed close to the Treiz ligament. Therefore, in order to prevent the future recurrence of IH, we retrospectively concluded that it was necessary to release the MD that was sutured closed in the initial surgery and suture the MD again at a part farther to the left side from the Treiz ligament. In our patients, the tissues around the hernia became hardened due to the postoperative changes, releasing the MD, which was sutured with barbed sutures, and was avoided as it would have risked damaging the blood vessels of the reconstructed intestine. However, we would like to emphasize again that to prevent hernia recurrence, it is better to re-close the MD.

The hernia orifice formed after suturing the MD is very narrow, so once the intestine protrudes into the orifice, it is prone to becoming incarcerated. This IH is more likely to develop into a serious condition than an IH without closing the MD; therefore, greater caution and immediate treatment are needed.

## CONCLUSIONS

IHs after MD closure are prone to incarceration and can easily become serious, so immediate treatment is needed. Although closure of the MD after B-II is necessary to prevent IH, it is recommended that the MD should be closed on the left side as far as possible from the duodenum situated at the Treiz ligament.

## ACKNOWLEDGMENTS

We would like to thank AJE (https://www.aje.com) for English language editing.

## DECLARATIONS

### Funding

No external support was obtained for this manuscript, including funding for equipment and drugs.

### Authors’ contributions

KM and TShim contributed to the acquisition of the clinical data and prepared the manuscript for this case report.

KM, KS, AM, TShim, NM, and TK performed the operations and perioperative management.

KM and TShim contributed to the drafting and revision of the manuscript.

TA, TShir, KG, and MM supervised decision-making, implemented the treatment plan for the patient, and prepared the manuscript.

All authors have read and approved the final version of the manuscript.

### Availability of data and materials

The data are not available for public access because of patient privacy concerns, but are available from the corresponding author on reasonable request.

### Ethics approval and consent to participate

The present study was conducted in accordance with the ethical standards of our institution. This work does not require ethical considerations or approval. Informed consent to participate in this study was obtained from the patients.

### Consent for publication

Informed consent was obtained from the patients for the publication of this case report and any accompanying images.

### Competing interests

The authors declare that they have no competing interests.
